# Retrotransposon expression in response to in vitro inoculation with two fungal pathogens of Scots pine (*Pinus sylvestris* L.)

**DOI:** 10.1186/s13104-019-4275-3

**Published:** 2019-04-29

**Authors:** Angelika Voronova

**Affiliations:** Genetic Resource Centre, Latvian State Forest Research Institute “Silava”, Rigas 111, Salaspils, 2169 Latvia

**Keywords:** Retrotransposon, Expression, Transcription, *Lophodermium seditiosum*, *Heterobasidion annosum*, Scots pine (*Pinus sylvestris* L.)

## Abstract

**Objective:**

Conifer genomes show high genetic diversity in intergenic regions that contain diverse sets of transposable elements with dominating long terminal repeat (LTR) retrotransposons (RE). Transcription of RE in response to environmental stimuli could produce various types of regulatory non-coding RNAs, but global genomic methylation changes could result in a coincidental expression of normally silent genomic regions. Expression of several RE families was evaluated in Scots pine seedlings after controlled inoculations with two fungal species that exhibit different modes of pathogenicity (necrotrophic and likely biotrophic); data compared to the overall RE distribution in genome. Recognition of regulatory non-coding RNA involved in host–pathogen interplay could be valuable in understanding defence mechanisms of perennial plants.

**Results:**

In the case of necrotrophic fungi *Heterobasidion annosum* (HA), short activation followed by restriction of RE expression was revealed after inoculation and during the spread of the pathogen. After inoculation with *Lophodermium seditiosum* (LS), an early increase in RE expression was revealed with the spread of the pathogen and subsequent transcription rise in all seedlings. Our observations indicate that in the complex plant genome multiple RE families constitutively express in response to pathogen invasion and these sequences could undergo regulation related to host response or pathogen influence.

**Electronic supplementary material:**

The online version of this article (10.1186/s13104-019-4275-3) contains supplementary material, which is available to authorized users.

## Introduction

Conifers belong to an ancient plant clade of gymnosperms, which are characterized by large continuous populations, an outcrossing pollination mode, long life cycle, strong adaptation to the environment and a smaller anthropogenic impact on the distribution of species. Conifer genomes are characterized by large, mostly diploid genomes (*P. sylvestris* (2C) = 46.96 pg [1]), which contain numerous repetitive sequences, pseudogenes, gene families and large inter-gene regions [2–4]. Up to 62% of the sequenced loblolly pine genome consists of RE sequences [3]. Compared to angiosperms, conifers contain diversified REs and fewer single LTRs [5]. In plants transcription and transposition of transposable elements is associated with stress conditions, meristematic tissues and certain stages in development [6, 7]. In a changing environment transposition and increased recombination rates could be an evolutionary tool for species adaptation [8–13]. However, expression of the RE does not directly imply further transposition. Large numbers of truncated elements are unable to transpose, but could contain regulatory motifs: transcription start sites, transcription factor binding sites, cis-acting elements, polyadenylation signals as well as methylation marks [12, 14]. Transposable elements were reported to be a source of microRNAs [15–17] and long non-coding RNAs [18] and that they could initiate transcription of antisense transcripts [19]. Pathogen–host interplay is an important force that drives the evolution of defence mechanisms in plants. In the current study a comparison of the transcriptional response to two important Scots pine fungal pathogens was performed: the necrotic fungi HA, the cause of root rot; and the less exterminating LS, the cause of seasonal needle cast in young trees. Relaxation of RE in response to fungal pathogens could reflect global chromatin methylation state changes in the host, a switch to stress responsive gene networks or the production of regulatory non-coding RNAs.

## Main text

### Methods

For the LS inoculation 27 grafted 2-year-old pine ramets from Ja3(5), Sm3(13) and Sm9(9) were used. One plant from each of the three genotypes was treated with water as a control. RNA was isolated from the needles of the same seedling before inoculation, 3 days post inoculation (dpi), 14 dpi and 31 dpi with prominent signs of infection. Details on LS inoculation are provided in (Additional file 1). For the HA inoculation 101 seedlings of 12 pine plus trees were used. 6-day-old healthy seedlings were inoculated with a HA suspension details provided in (Additional file 2). 2–4 seedlings from each family were used as non-inoculated controls and harvested after 7, 14 and 21 dpi. For each seedling RNA was isolated from the roots and shoots separately. Samples from both experiments were stored at − 80 °C until extraction.

RNA was isolated using the method described by [20]. Treatment with the Turbo DNA-free kit (*Thermo Fisher Scientific*) was performed for all samples following the manufacturer’s instructions. RNA purity was tested by polymerase chain reaction (PCR) with an RNA stock solution as a template and oligonucleotides amplifying REs and *APT1*. RNA concentration was measured with a Qubit (*Life technologies*) and equated. The Taqman Reverse transcription kit (*Applied Biosystems*) was used for reverse transcription of 0.8 μg RNA with random hexamer oligos (*Thermo Fisher Scientific*). Produced cDNA was diluted 1:4 with nuclease-free water. Primers to 12 RE polyprotein sequences [29] were used, expression levels of the antimicrobial gene *Pinosylvin synthase* (*PsBs*) was evaluated to compare the induction of defence responses. Comparative CT real-time PCR was performed with the SyberGreen Maxima qPCR Master Mix (*Thermo Fisher Scientific*) standard protocol on a StepOnePlus thermocycler (*Thermo Fisher Scientific*). For each sample two technical replications per plate were used in order to analyse more biological replicates and to include three reference genes. The normalization coefficient was calculated and a correction applied for plates with identical sample sets [21], identical thresholds for each target were set. Primers for pine endogenous reference genes were designed using Primer3 v.4.1.0. software [22], (Additional file 3). NormFinder [23] and Bestkeeper [24] were used to evaluate the most stable genes. For the evaluation of the amplification efficiencies, the standard curve method was used: qPCR with six 1:10 dilutions of the experimental cDNA were performed in triplicate and the efficiency was calculated using StepOne Software v.2.2.2 (*Applied Biosystems*). The relative expression level (ΔΔCt) of REs before and after inoculation was calculated [25]. The amplification product melting curves provided in (Additional file 4). Multi-Response Permutation Procedures (MRPP) provided by PC-ORD v.5. statistical package [26] was used to investigate differences between expression responses after HA. Pearson correlation was calculated for the shoots and roots HA data on four RE loci and phenotype observed.

### Results

#### Inoculation with LS

**Fig. 1:**
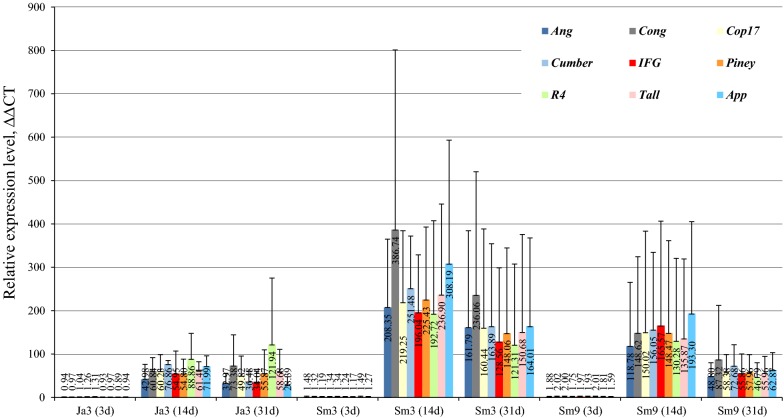
RE relative expression across the ramets of three pine clones (Ja3, Sm3, Sm9) after inoculation with LS. Tissue type: needles. Control trees exhibited no RE expression at time points studied and therefore the data are not shown (Additional file 5a). Ang: *Angelina* RE; Cumber: *Cumberland* RE; R4: *Riga*-*4* RE; Cong: *Conagree* RE; IFG: *IFG*-*7* RE; Tall: *Talladega* RE; Cop17: *Copia*-*17_PAb* RE; Piney: *Pineywoods* RE; App: *Appalachian* RE

No *PsBs* or REs expression level changes were observed at all-time points studied in the healthy controls (Additional file 5a). Relative *PsBs* expression induction was detected at 3 dpi and expression levels increased as disease progressed (Additional file 5b). At 14 dpi and 31 dpi following inoculation with LS all tested families of REs were expressed at high levels, but *PsBs* and RE expression correlated (Additional file 6a). The three studied genotypes showed varying RE expression responses at the sampled time points. On average Sm3 ramets showed higher RE expression and earlier induction at all-time points (Fig. 1). REs *Conagree* and *Appalachian* were expressed more intensively in Sm3; *Appalachian* expression was higher in Sm9, but in Ja3 *Riga*-*4* was expressed more intensively. An observed increase in RE expression level was confirmed by absolute transcript quantification of the *Conagree* element as described previously [27], (Additional file 5c).

#### Inoculation with HA

Following signs of infection were observed in inoculated seedlings compared to the control seedlings: reduced length of the inoculated roots, absence or reduced number of lateral rootlets, reduced number and length of secondary needles, and yellowing of needles and stem (Additional file 7a). In all seedlings at 7 dpi the enhancement of *PsBs* levels was observed mostly in the roots. At 14–21 dpi *PsBs* expression was increased in the shoots (Additional file 7b). The levels of *PsBs* expression in families M259, M248, M110, M241 were lower compared to other seedling families at each time point studied.

**Fig. 2:**
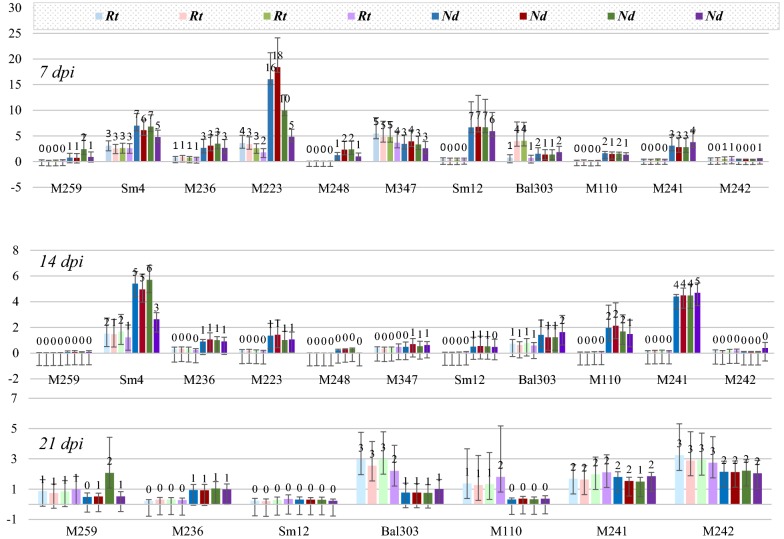
Relative expression of REs after HA inoculation at 7 dpi, 14 dpi and 21 dpi in Rt (roots) and Nd (shoots), overall mean per seedling family (3–4 seedlings)

**Fig. 3:**
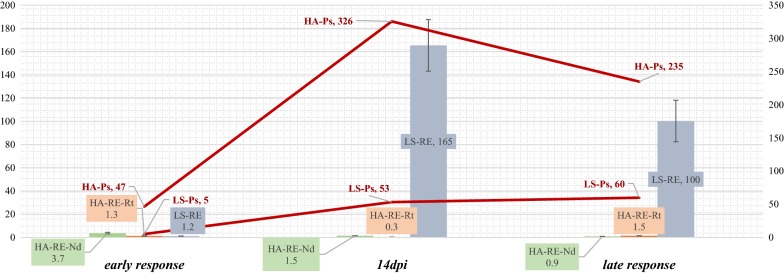
Summary of the RE and gene PsBs (Ps, *Pinosylvin synthase*) expression (overall mean) after inoculation with LS and HA. Nd-needles; Rt-roots. *Ps* (*Pinosylvin synthase* expression, red lines); RE (retrotransposon expression); LS (inoculation with *Lophodermium seditiosum*); HA (inoculation with *Heterobasidion annosum*, Nd-shoots; Rt-roots); numbers indicate overall mean of expression (∆∆Ct) relative to healthy controls

For Sm12 and M110 seedling families, the expression of nine REs was analysed (Additional file 7c). No RE expression enhancement in roots and a small induction of RE expression at 7 dpi was revealed. The M110 family displayed a lower induction of REs at 7 dpi and 14 dpi. At 21 dpi no RE expression was observed in either tissues. Since enhancement of different RE loci in one seedling correlates strongly (Additional file 6b), four representative REs and *PsBs* were selected to perform qPCR analyses on remaining seedlings. A more diversified response was observed following the analysis of 11 pine seedling families (Fig. 2). RE expression was enhanced in the shoots at 7 dpi in all seedling families tested. At 14 dpi in some seedlings RE expression was increased in the shoots, but at 21 dpi RE expression in the shoots was decreased while a small increase was observed in the roots. Overall levels of RE transcription were significantly lower after inoculation with HA when compared to the response after LS inoculation (Fig. 3).

#### Analysis of groupings & correlation

MRPP of groups by treatment time indicated significant difference between the control group and each sampling point after infection, while heterogeneity between groups taken after 7 dpi, 14 dpi, 21 dpi were similar to that expected by chance (Additional file 8). In correlation analysis, RE expression in roots weakly correlated with stem damage, but RE expression in needles weakly correlated with infection state. A strong correlation (0.998) was found between the expression of all RE loci in each tissue type (Additional file 9a). Correlation between RE expression in shoot and root tissues within five particular seedling families (Sm4, M236, M223, M248, M347) was found to be strong to moderate (Additional file 9b). In most analysed pine seedling families *PsBs* gene expression seemed to occur independently from the RE loci, except M259, Sm4, M236 families where RE expression correlated with *PsBs* expression in the opposite tissue at weak to moderate levels.

### Discussion

Although activation of RE in response to stress has been shown in many plants [28–31], usually transposition of autonomous elements were studied [32–34]. Meanwhile, non-autonomous REs are transposed in plants [35, 36], but transcription could also occur in disrupted sequences, which could contribute to non-coding RNA production [18, 37]. RE in conifers was characterized as more diverse and abundant when compared to angiosperm repeats [5]. For example, in the tomato genome the active *TLC1.1* element is found in 900 copies and this RE is a dominating family [38]. In the genome of *Pinus taeda* about 1500 RE families were revealed [5]. Random relaxation of thousands of RE families found in the large genome would result in the neutral enhancement of the most distributed RE families. However, the results obtained in this study demonstrate no connection between RE copy number distribution in the genome and relative levels of transcriptional induction. The most abundant in terms of copy numbers per single diploid genome of *Pinus sylvestris* from RE studied was *IFG* (83,986 ± 7439) first detected in sugar pine [39]. Nevertheless, *Appalachian*, *Riga*-*4* and *Cumberland* transcripts were found to be more abundant than *IFG* transcripts in both the HA and LS experiments. *Appalachian* was found to be propagated in the *P. sylvestris* genome with 22,885 ± 3158 copies; *Riga*-*4* with 3916 ± 686 copies; *Cumberland* RE only with 165 ± 16.68 copies [27].

LS is an ascomycetous foliar inhabiting fungi and is a serious pathogen of the young trees of *P. sylvestris*, where it causes needle cast [40–43]. Pine sprouts with secondary needles are able to survive single LS attacks and develop new healthy shoots in the next season (Lagerberg 1913, cited by [40]). Most *Lophodermium* species are endophytes and pathogenicity has likely evolved via endophysm in this group [44]. *L. nitrenes* and *L. pinastri* were found to be propagated in needles as a slowly growing biotrophic fungi that avoids penetration into mesophyll cells during the early stages of infection [45, 46]. Scots pine originated from Latvian seed orchard *Smiltene* were carrying induced LS defence and recovery rate [47]. HA is an aggressive pathogen belonging to basidiomycete necrotrophic fungi that colonize stumps and roots [48]. Necrotrophic fungi secrete cell-wall-degrading enzymes and toxins, which they use for rapid propagation in dead cells through induction of host plant hypersensitive responses [49, 50]. Defence against biotrophs and necrotrophs in *Arabidopsis* involve different Jasmonic acid/Ethylene-dependent or Salicylic acid-dependent pathways [51]. For inoculation with HA, no significant increase in RE expression was evaluated, which was in contrast to the response after inoculation with LS. Differences in RE response were observed for the pine seedling families, while gene *PsBs* expression increased constantly with the spread of HA and LS. In the current study it was shown that sets of REs responded to both fungi pathogens, but the levels and patterns of their responses was considerably different and more prominent in likely biotrophic pathogen inoculations. In more aggressive necrotrophic HA infections, RE transcription levels were significantly lower at time points and tissues studied.

There is growing evidence regarding the role of non-coding RNA in host–pathogen interplay through different RNA interference mechanisms [18, 52]. TEs could be acquired by horizontal gene transfer [53] and pathogenic strategies could rapidly evolve. Advantageous flexible stress responsive gene networks could be formed using REs, contrary, some pathogens could acquire features that through the agency of host REs downregulates transcription of nearby genes by various mechanisms. Evolutionary change via recombination, transposition and/or fast degradation of influenced RE sequences result in high genetic diversity and could induce adaptability of populations that undergoes continuous and variable pressure of the natural selection.

## Limitations

Major limitations are connected to genetic diversity of pine seedlings, individual variation was high as expected for a species with open cross-pollination and highly variable RE loci. Due to the uneven dispersion of the inoculum of LS some needles might acquire more hyphae accidentally. This could result in differences in the slowly growing LS propagation in different needles. Additionally, differing rootstock genotypes might have a considerable impact on expression in individual ramets [30].

## Additional files

**Additional file 1.** LS inoculation.

**Additional file 2.** *In vitro* inoculation with HA culture suspension.

**Additional file 3.** Reference gene primers used for normalization and control.

**Additional file 4.** Melting curves of amplified targets.

**Additional file 5.** **a.** Relative expression of 9 retrotransposon families across the ramets of three pine clones (Ja3, Sm3, Sm9) after inoculation with LS. Tissue type: needles. **b.** *PsBs* antimicrobial gene mean relative expression in Ja3, Sm9 pine ramets after LS inoculation (3dpi, 14dpi and 31dpi). **c.** Mean and overall mean *Conagree* transcript copy numbers after LS inoculation, absolute quantification.

**Additional file 6.** **a.** Scatterplot matrix for pooled RE expression data correlation in needles after inoculation with LS. **b.** Scatterplot matrix for pooled RE expression data correlation in needles and roots after inoculation with HA.

**Additional file 7.** **a.** Matrix of observed traits and mean relative expression level of four RE loci and antimicrobial PsBs (*Pinosylvin synthase*) gene after inoculation with HA among pine seedlings root and shoot tissues. **b.** Mean relative expression level of the antimicrobial gene *PsBs* (*Pinosylvin synthase*) after inoculation with HA among pine seedlings studied in root (SAK) and shoot (SKU) tissues. **c.** Expression of nine REs after HA inoculation in seedling families Sm12 and M110 in shoot and root tissues.

**Additional file 8.** Summary of experimental group MRPP statistics.

**Additional file 9.** **a.** All-to-all correlation between expression data and observed damages after inoculation with HA. **b.** Correlation between expression data within pine seedling families after inoculation with HA.

## Abbreviations

LTR: long terminal repeat
REs: retrotransposons
HA: *Heterobasidion annosum*
LS: *Lophodermium seditiosum*
dpi: days post inoculation
*PsBs*: Pinosylvin synthase gene
MRPP: Multi-Response Permutation Procedures

## References

[1] Fuchs J, Jovtchev G, Schubert I (2008). The chromosomal distribution of histone methylation marks in gymnosperms differs from that of angiosperms. Chromosom Res..

[2] Nystedt B, Street NR, Wetterbom A, Zuccolo A, Lin Y-C, Scofield DG (2013). The Norway spruce genome sequence and conifer genome evolution. Nature.

[3] Zimin A, Stevens KA, Crepeau MW, Holtz-Morris A, Koriabine M, Marcais G (2014). Sequencing and assembly of the 22-gb loblolly pine genome. Genetics.

[4] Stevens KA, Wegrzyn JL, Zimin A, Puiu D, Crepeau M, Cardeno C (2016). Sequence of the sugar pine megagenome. Genetics.

[5] Wegrzyn JL, Liechty JD, Stevens KA, Wu LS, Loopstra CA, Vasquez-Gross HA (2014). Unique features of the loblolly pine (*Pinus taeda* L.) megagenome revealed through sequence annotation. Genetics..

[6] Wessler SR (1998). Transposable elements and the evolution of gene expression. Symp Soc Exp Biol.

[7] Martínez G, Slotkin RK (2012). Developmental relaxation of transposable element silencing in plants: functional or byproduct?. Curr Opin Plant Biol.

[8] Wessler SR, Bureau TE, White SE (1995). LTR-retrotransposons and MITEs: important players in the evolution of plant genomes. Curr Opin Genet Dev.

[9] Grandbastien M-A, Lucas H, Morel J-B, Mhiri C, Vernhettes S, Casacuberta J (1997). The expression of the tobacco Tnt1 retrotransposon is linked to plant defense responses. Genetica.

[10] Baucom RS, Estill JC, Leebens-Mack J, Bennetzen JL (2009). Natural selection on gene function drives the evolution of LTR retrotransposon families in the rice genome. Genome Res.

[11] Bennetzen JL (2000). Transposable element contributions to plant gene and genome evolution. Plant Mol Biol.

[12] Feschotte C (2008). The contribution of transposable elements ot the evolution of regulatory networks. Nat Rev Genet.

[13] Galindo-González L, Mhiri C, Deyholos MK, Grandbastien MA (2017). LTR-retrotransposons in plants: engines of evolution. Gene.

[14] Slotkin RK, Martienssen R (2007). Transposable elements and the epigenetic regulation of the genome. Nat Rev Genet.

[15] Piriyapongsa J, Jordan IK (2008). Dual coding of siRNAs and miRNAs by plant transposable elements. RNA.

[16] Ito H (2013). Small RNAs and regulation of transposons in plants. Genes Genet Syst..

[17] Li Y, Li C, Xia J, Jin Y (2011). Domestication of transposable elements into MicroRNA genes in plants. PLoS ONE.

[18] Wang D, Qu Z, Yang L, Zhang Q, Liu ZH, Do T (2017). Transposable elements (TEs) contribute to stress-related long intergenic noncoding RNAs in plants. Plant J..

[19] Zinad HS, Natasya I, Werner A (2017). Natural antisense transcripts at the interface between host genome and mobile genetic elements. Front Microbiol..

[20] Rubio-Piña JA, Zapata-Pérez O (2011). Isolation of total RNA from tissues rich in polyphenols and polysaccharides of mangrove plants. Electron J Biotechnol..

[21] D’haene B, Vandesompele J, Hellemans J (2010). Accurate and objective copy number profiling using real-time quantitative PCR. Methods..

[22] Untergasser A, Cutcutache I, Koressaar T, Ye J, Faircloth BC, Remm M (2012). Primer3–new capabilities and interfaces. Nucleic Acids Res.

[23] Andersen CL, Jensen JL, Orntoft TF (2004). Normalization of real-time quantitative reverse transcription-PCR data: a model-based variance estimation approach to identify genes suited for normalization, applied to bladder and colon cancer data sets. Cancer Res.

[24] Pfaffl MW, Tichopad A, Prgomet C, Neuvians TP (2004). Determination of stable housekeeping genes, differentially regulated target genes and sample integrity: BestKeeper–Excel-based tool using pair-wise correlations. Biotechnol Lett.

[25] Pfaffl MW (2001). A new mathematical model for relative quantification in real-time RT-PCR. Nucleic Acids Res.

[26] McCune B, Mefford MJ (2006). PC-ORD multivariate analysis of ecological data version 5.10.

[27] Voronova A, Belevich V, Korica A, Rungis D (2017). Retrotransposon distribution and copy number variation in gymnosperm genomes. Tree Genet Genomes..

[28] McClintock B (1984). The significance of responses of the genome to challenge. Science..

[29] Wessler SR (1996). Plant retrotransposons: turned on by stress. Curr Biol.

[30] Grandbastien MA (2015). LTR retrotransposons, handy hitchhikers of plant regulation and stress response. Biochim Biophys Acta Gene Regul Mech..

[31] Capy P, Gasperi G, Biémont C, Bazin C (2000). Stress and transposable elements: co-evolution or useful parasites?. Heredity (Edinb)..

[32] Butelli E, Licciardello C, Zhang Y, Liu J, Mackay S, Bailey P (2012). Retrotransposons control fruit-specific, cold-dependent accumulation of anthocyanins in blood oranges. Plant Cell..

[33] Rocheta M, Carvalho L, Viegas W, Morais-Cecílio L (2012). Corky, a gypsy-like retrotransposon is differentially transcribed in Quercus suber tissues. BMC Res Notes..

[34] Matsunaga W, Ohama N, Tanabe N, Masuta Y, Masuda S, Mitani N (2015). A small RNA mediated regulation of a stress-activated retrotransposon and the tissue specific transposition during the reproductive period in Arabidopsis. Front Plant Sci..

[35] Witte C-P, Le QH, Bureau T, Kumar A (2001). Terminal-repeat retrotransposons in miniature (TRIM) are involved in restructuring plant genomes. Proc Natl Acad Sci.

[36] Kalendar R, Vicient CM, Peleg O, Anamthawat-Jonsson K, Bolshoy A, Schulman AH (2004). Large retrotransposon derivatives: abundant, conserved but nonautonomous retroelements of barley and related genomes. Genetics.

[37] Kapranov P, St. Laurent G (2012). Dark matter RNA: existence, function, and controversy. Front Genet..

[38] Tapia G, Verdugo I, Yañez M, Ahumada I, Theoduloz C, Cordero C (2005). Involvement of ethylene in stress-induced expression of the TLC1.1 retrotransposon from *Lycopersicon chilense* Dun. Plant Physiol..

[39] Kossack DS, Kinlaw CS (1999). IFG, a gypsy-like retrotransposon in Pinus (Pinaceae), has an extensive history in pines. Plant Mol Biol.

[40] Testing Martinsson O (1979). Scots pine for resistance to Lophodermium needle cast.

[41] Gregory SC, Redfern D (1998). Disease and disorders of forest trees Forestry C.

[42] Minter DW (1981). Lophodermium on Pines. Mycol Pap..

[43] Rack K (1963). Studies on needle-cast of Scots pine I-III. 2. Pflanz Krankheiten..

[44] Ortiz-García S, Gernandt DS, Stone JK, Johnston PR, Chapela IH, Salas-Lizana R (2003). Phylogenetics of Lophodermium from pine. Mycologia.

[45] Deckert RJ, Melville LH, Peterson RL (2001). Structural features of a Lophodermium endophyte during the cryptic life-cycle phase in the foliage of *Pinus strobus*. Mycol Res.

[46] Ponge J (2010). The soil under the microscope: the optical examination of a small area of Scots pine litter.

[47] Jansons A, Neimane U, Baumanis I (2008). Needlecast resistance of Scots pine and possibilities of its improvement. Mezzinatne..

[48] Asiegbu FO, Adomas A, Stenlid J (2005). Conifer root and butt rot caused by *Heterobasidion annosum* (Fr.) Bref. s.l. Mol Plant Pathol..

[49] Govrin EM, Levine A (2000). The hypersensitive response facilitates plant infection by the necrotrophic pathogen *Botrytis cinerea*. Curr Biol.

[50] Nagy ED, Lee T-C, Ramakrishna W, Xu Z, Klein PE, SanMiguel P (2007). Fine mapping of the Pc locus of *Sorghum bicolor*, a gene controlling the reaction to a fungal pathogen and its host-selective toxin. Theor Appl Genet.

[51] Thomma BP, Eggermont K, Penninckx IA, Mauch-Mani B, Vogelsang R, Cammue BP (1998). Separate jasmonate-dependent and salicylate-dependent defense-response pathways in Arabidopsis are essential for resistance to distinct microbial pathogens. Proc Natl Acad Sci USA..

[52] Weiberg A, Jin H (2015). Small RNAs—the secret agents in the plant–pathogen interactions. Curr Opin Plant Biol.

[53] Panaud O (2016). Horizontal transfers of transposable elements in eukaryotes: the flying genes. C R Biol..

