# Supplementary material for: Retrotransposon expression in response to in vitro inoculation with two fungal pathogens of Scots pine (Pinus sylvestris L.)
Source: BMC Res Notes. 2019 Apr 29;12:243. doi: 10.1186/s13104-019-4275-3 (PMC6489336; doi:10.1186/s13104-019-4275-3)
Supplement: Supplementary file 1 — Additional file 1. LS inoculation. [file 13104_2019_4275_MOESM1_ESM.docx]

**Additional** **file 1**. a) Two-year-old grafted *P. sylvestris* clones were used in the *Lophodermium seditiosum* (LS) inoculation experiment*. b) Scots pine ramets one month after inoculation with LS.

*
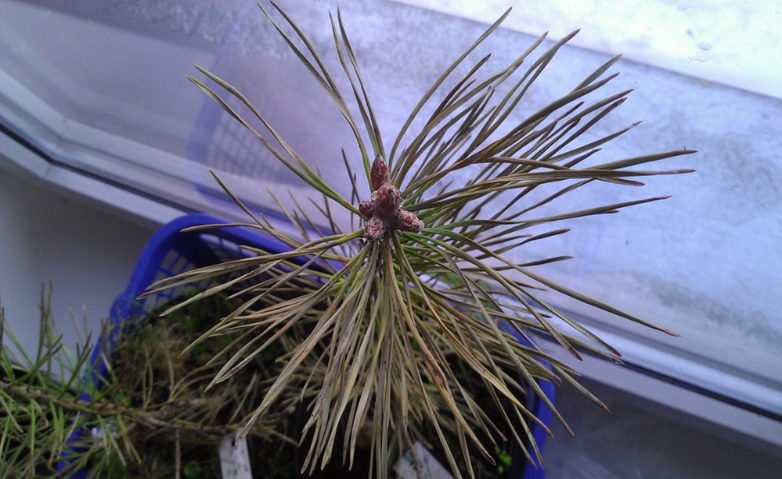

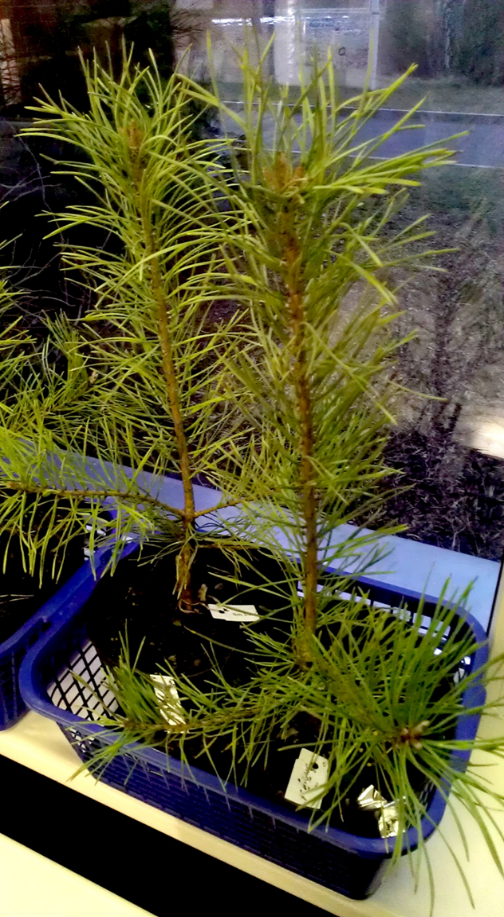
*

**a)**

**b)**

*Local LS isolate *PS014201* and the propagation protocol was obtained from the Unit of Plant Pathology and Entomology of the Institute of Horticulture in personal communication with Inga Moročko-Bičevska. The LS isolate was used for culture propagation in 200 mL liquid MEB medium for 3 weeks at +18°C and 120 rpm in an OS-20 orbital shaker (*Biosan*). The culture was centrifuged and rinsed three times with distilled water, after which it was homogenized in 30 mL water using a sterile LabGen-7 homogenizer (*Coleparmer*). 15 mL of the concentrated culture suspension was diluted to 100 mL with dH_2_O and plants were sprayed equally. After inoculation all plants were covered for 3 days with transparent polyethylene film and additionally sprayed daily with dH_2_O to promote wet conditions favourable for pathogen growth. Control trees from each ramet were treated and sampled in the same way but only sprayed with water. The first brown spots on needles were observed at 3 days post inoculation (dpi), while the control trees were healthy. Needles with equal amount of spots were selected for analysis at each time point and stored in liquid nitrogen until extraction. LS in liquid medium tend to form colonies and the homogenization method used might not disperse thoroughly the inoculum (as proved by microscopy) and therefore due to the uneven dispersion of the inoculum some needles might acquire more hyphae accidentally. This could result in slight differences in the slowly growing LS propagation on different needles. Additionally, differing rootstock genotypes might have a considerable impact on expression in individual ramets [30].
