# Supplementary material for: Retrotransposon expression in response to in vitro inoculation with two fungal pathogens of Scots pine (Pinus sylvestris L.)
Source: BMC Res Notes. 2019 Apr 29;12:243. doi: 10.1186/s13104-019-4275-3 (PMC6489336; doi:10.1186/s13104-019-4275-3)
Supplement: Supplementary file 2 — Additional file 2. In vitro inoculation with HA culture suspension. [file 13104_2019_4275_MOESM2_ESM.docx]

**Aditional file 2.** a) Example of *in vitro* inoculation with *Heterobasidion annosum* (HA) culture suspension. b) Scots pine seedlings of one family (Sm12)*. The first 4 tubes contain non-infected controls; next 6 tubes contain seedlings inoculated with HA** after 21 dpi.

**b)**


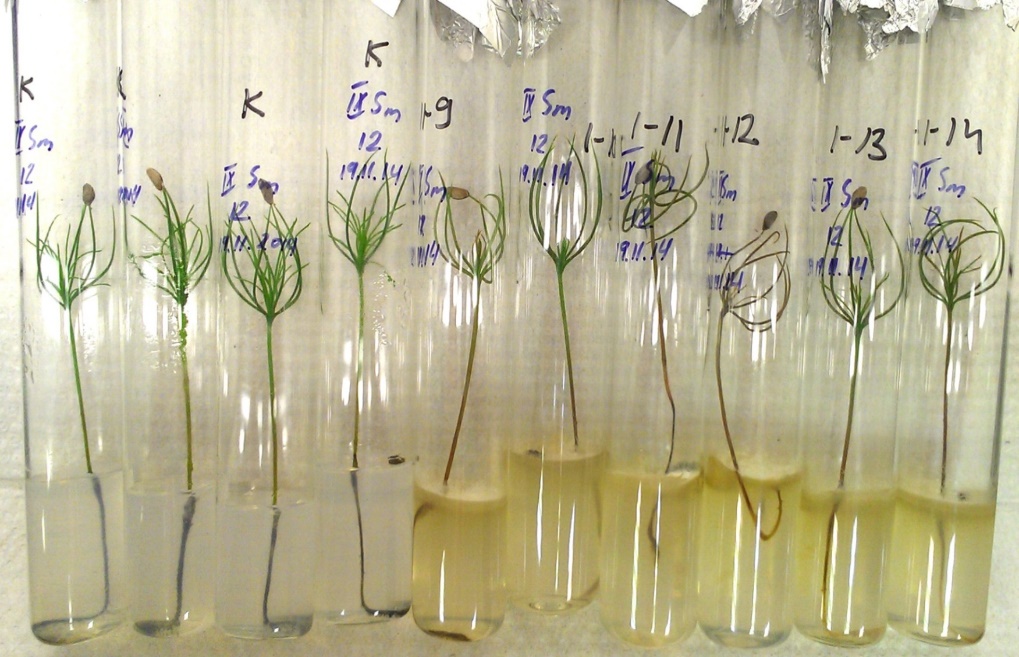

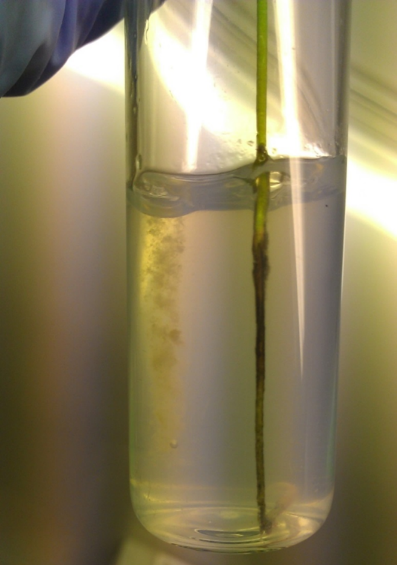


**a)**

**b)**

*Plus trees originating from different sites in Latvia are indicated by the first letter: M-Misa, Bal-Baldone, Sm-Smiltene (M223, M347, M242, M259, M110, M248, M241, M236, M247, Sm12, Sm4, Bal303). Seeds were sterilized by soaking for 10 minutes in 96% ethanol in a TopSafe biological safety cabinet and after evaporation of the ethanol the seeds were placed in sterile glass tubes with 0.5% MS medium, sealed and placed in a growing chamber.

**A local isolate of HA was received from the LSFRI Silava Forest Phytopathology and Mycology Laboratory. The HA culture was propagated in MEB liquid medium in petri dishes at +18°C for a 2 weeks before the experiment. The inoculation suspension was prepared as follows: HA cultures from petri dishes were transferred to a 50 mL falcon tube, rinsed with distilled water and centrifuged for 2 mins at 6000 rpm (repeated 3 times). In the final stage 20 mL dH_2_O was added and the culture was crushed using a sterile LabGen-7 homogenizer (*Coleparmer*). Liquid suspensions were combined in one tube. Seedlings were inoculated with 50 μL of the HA culture suspension by dipping them into the medium to approx. 0.5 cm away from the seedling root. 2-4 seedlings from each family were harvested after 7, 14 and 21 dpi. 2-4 seedlings from each family were used as non-inoculated controls. For each seedling RNA was isolated from the roots and shoots and analysed separately. Inoculation with HA was indirect, meaning that fungi should first regrow in the medium and only then establish contact with undamaged seedlings. At 21 dpi, the following signs of infection were observed in inoculated seedlings compared to the control seedlings: reduced length of the inoculated roots, absence or reduced number of lateral rootlets, reduced number and length of secondary needles, and yellowing of needles and stem.
