# Supplementary material for: Retrotransposon expression in response to in vitro inoculation with two fungal pathogens of Scots pine (Pinus sylvestris L.)
Source: BMC Res Notes. 2019 Apr 29;12:243. doi: 10.1186/s13104-019-4275-3 (PMC6489336; doi:10.1186/s13104-019-4275-3)
Supplement: Supplementary file 3 — Additional file 3. Reference gene primers used for normalization and control. [file 13104_2019_4275_MOESM3_ESM.docx]

| **Nr.** | **Name** | **Sequence (5'->3')** | **Product, bp** | **Amplification efficiency** | **Reference/ Gene bank accession number** |
| --- | --- | --- | --- | --- | --- |
| 1 | UBI_F | GAAGGAGCAGTGGAGTCCTG | 104 | 1.0935 | [1] |
|  | UBI_R | CAATTTCAGGGACGAGAGGA |  |  |  |
| 2 | rRNA18S_F | TTCTGCCCTATCAACTTTCG | 112 | - | [2] |
|  | rRNA18S_R | GATGTGGTAGCCGTTTCTCA |  |  |  |
| 3 | GAP_F | ACGGTTTTGGTCGAATTGGA | 70 | 1.1424 | [3] |
|  | GAP_R | CCCCACGAGCTCGATATCAT |  |  |  |
| 4 | aTub_F | GGTTGGAAATGCTTGCTGGG | 106 | - | HE632838.1, HE637278.1, AM983412.1, KF158844.1 |
|  | aTub_R | GTTGAATGCATCGTCTCCGC |  |  |  |
| 5 | EF1α_F | GGGAAGCCACCCAAAGTTTT | 160 | 1.03836 | [4] |
|  | EF1α_R | TACATGGGAAGACGCCGAAT |  |  |  |
| 6 | UEP_F | GGTCAAGAGGCTGAGGAAGG | 102 | 1.15031 | HE638578.1, HE627067.1, HE638519.1 |
|  | UEP_R | GTAAGCCCACACTTGCCACA |  |  |  |
| 7 | APT1_F | GGCACCTTATCTGCAGCCAT | 106 | 1.1455 | NM_179383.2,  BT113945.1, BT071219.1, GT260896.1, GT258063.1 |
|  | APT1_R | CTAACTTCTCCCGGCCCTT |  |  |  |
| 8 | PsBs_F | AAGAGAGTGGGCGTGTTCCA | 72 | - | X60753.1,  [3] |
|  | PsBs_R | GGCAAGGTCTTTCGCCATT |  |  |  |

**Additional file 3.** Primers tested for the stability value* for use as the endogenous controls. Antimicrobial gene *PsBs* (Pinosylvin synthase or pine stilbene synthase) was used as the control of the defence response induction.

* The stability values of seven reference genes were analysed for each treatment and tissue type: ubiquitin, *UBI*; 18S ribosomal RNA, *rRNA18S*; glyceraldehyde-3-phosphate dehydrogenase, *GAPDH*; α-tubulin, *aTUB*; elongation factor 1α, *EF1a*; ubiquitin extension protein, *UEP*; adenine phosphoribosyl transferase 1, *APT1*. *GAPDH*, *EF1a* and *UEP* were used as reference genes for the LS experiment as according to Normfinder the gene stability value was the lowest for *GAP* (0.015) and *UEP* (0.016); however, Bestkeeper showed *EF1a* to be the most stable reference. Best stability values for HA-inoculated root tissue were for the UBI gene (0.084) or the two-gene combination of EF1a and UBI (0.104). For HA-inoculated shoot tissue, the most stable were APT1 (0.006) or the combination of APT1 and UBI (0.029). Additionally, GAPDH was used in combination with these reference genes, as with other software (Bestkeeper) it was found to be one of the most stable genes in both tissues and was used for the mutual normalization of different plates.

References:

1. Vuosku J, Sarjala T, Jokela A, Sutela S, Sääskilahti M, Suorsa M, *et al.* One tissue, two fates: Different roles of megagametophyte cells during Scots pine embryogenesis. J Exp Bot. 2009;60(4):1375–1386.
2. Gonçalves S, Cairney J, Maroco J, Oliveira MM, Miguel C. Evaluation of control transcripts in real-time RT-PCR expression analysis during maritime pine embryogenesis. Planta. 2005;222(3):556–563.
3. Skipars V, Krivmane B, Rungis D. Thaumatin–like protein gene copy number variation in Scots pine (*Pinus sylvestris*). Environ Exp Biol. 2011;9:75–81.
4. Hirao T, Fukatsu E, Watanabe A. Characterization of resistance to pine wood nematode infection in Pinus thunbergii using suppression subtractive hybridization. BMC Plant Biol. 2012;12(1):13.
