# Supplementary material for: Retrotransposon expression in response to in vitro inoculation with two fungal pathogens of Scots pine (Pinus sylvestris L.)
Source: BMC Res Notes. 2019 Apr 29;12:243. doi: 10.1186/s13104-019-4275-3 (PMC6489336; doi:10.1186/s13104-019-4275-3)
Supplement: Supplementary file 4 — Additional file 4. Melting curves of amplified targets. [file 13104_2019_4275_MOESM4_ESM.docx]

**Additional file 4.** Templates amplification product melting curves. Endogenous controls: *rRNA18S* (18S ribosomal RNA), *GAPDH* (glyceraldehyde-3-phosphate dehydrogenase), *αTUB* (α-tubulin), *UBI* (ubiquitin), *EF1α* (elongation factor 1α), *UEP* (ubiquitin extension protein), *APT1* (adenine phosphoribosyl transferase); control gene *PsBs* (Pinosylvin synthase or pine stilbene synthase); retrotransposons: *Silava_PTa*, *Copia-17*, *ptAngelina*, *ptBastroop*, *ptConagree*, *ptOuachita*, *ptOzark*, *ptPineywoods*, *PtTalladega*, *IFG*, *PsAppalachian*, *PtCumberland* and *Riga-4*.


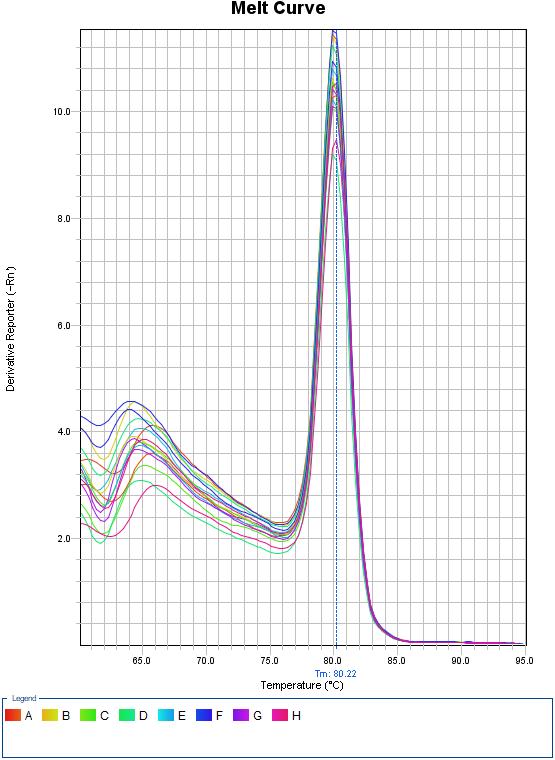


rRNA18S


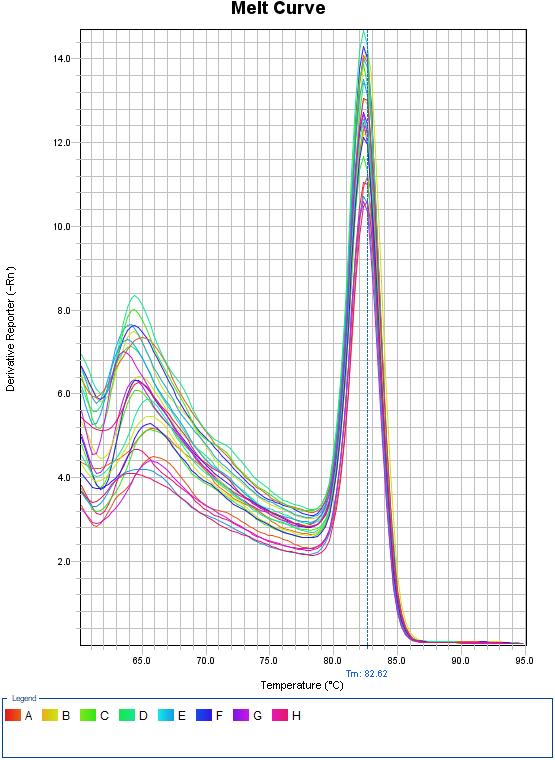


αTub


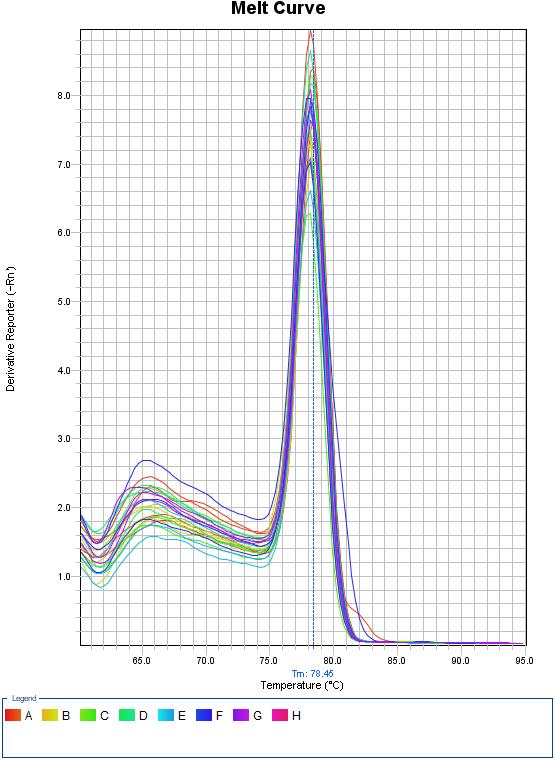


UBI


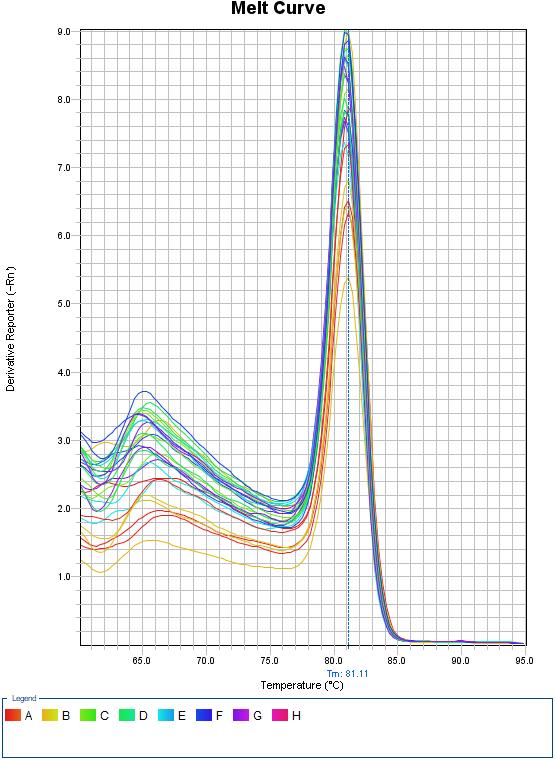


GAPDH


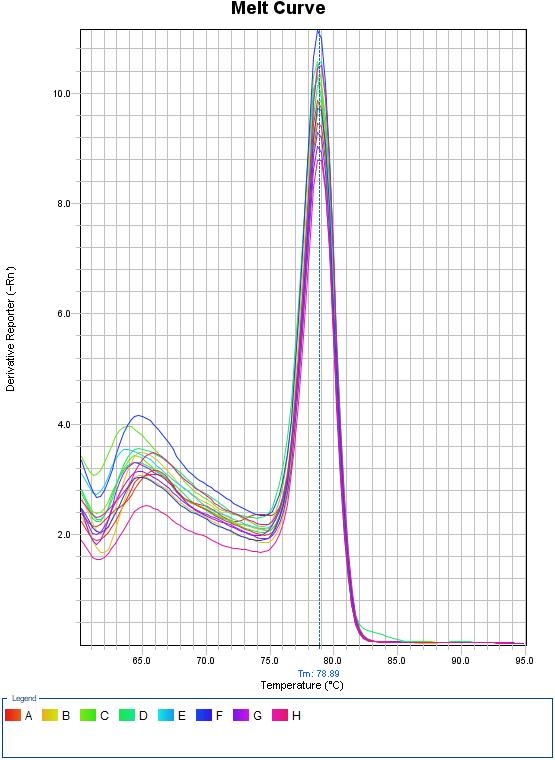


APT1


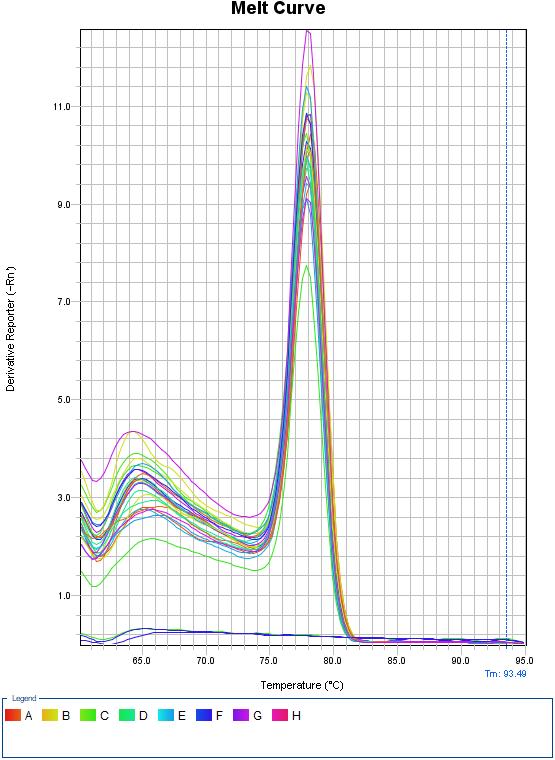


EF1α


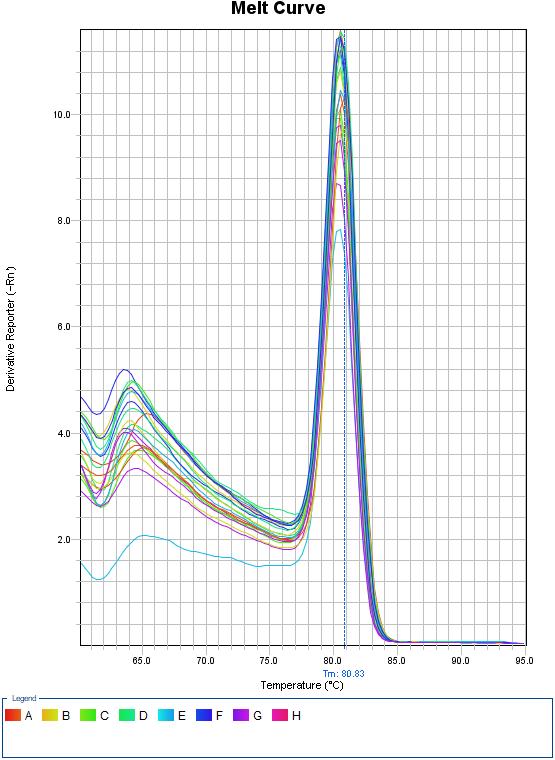


UEP


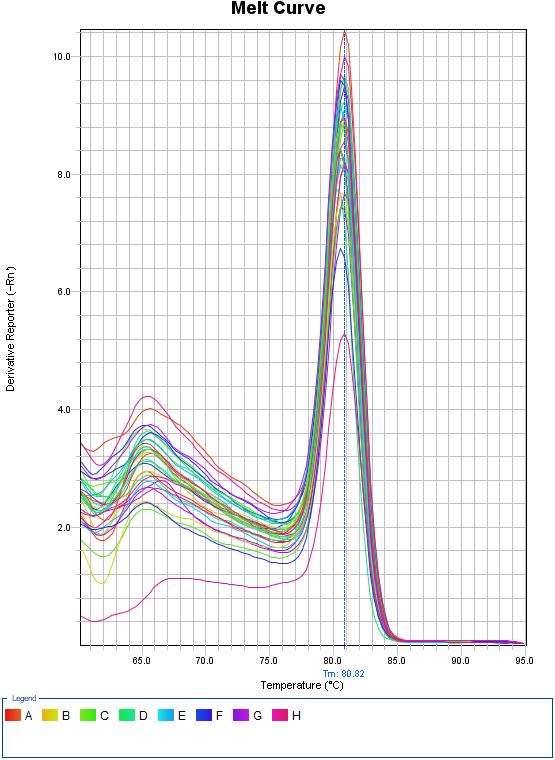


PsBs


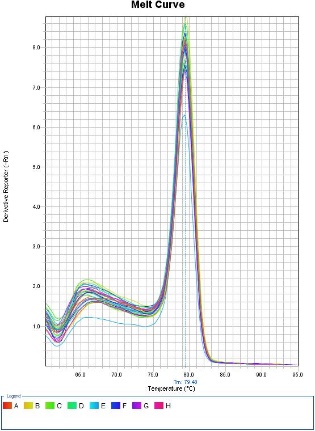


*Angelina*


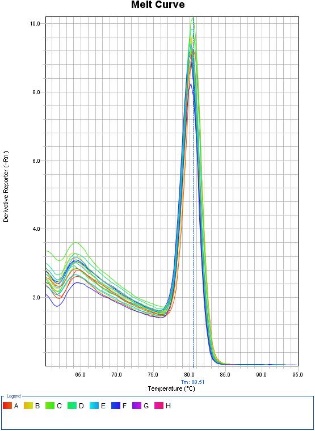


*Appalachian*


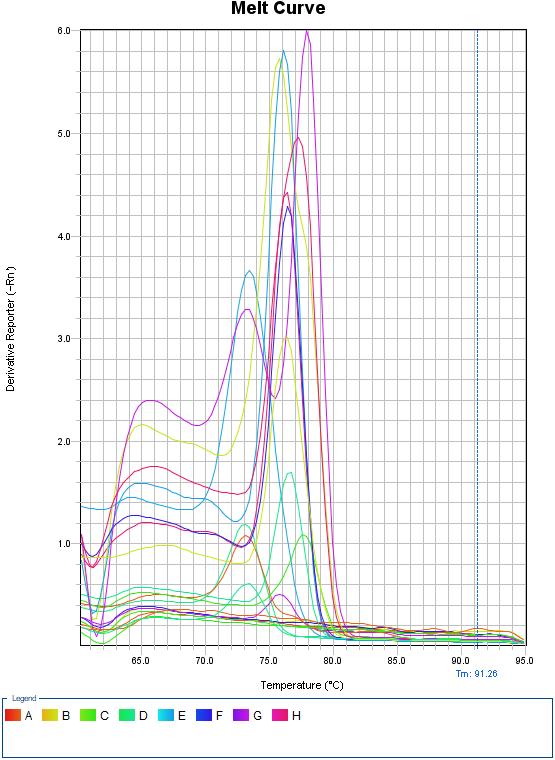


*Bastroop**


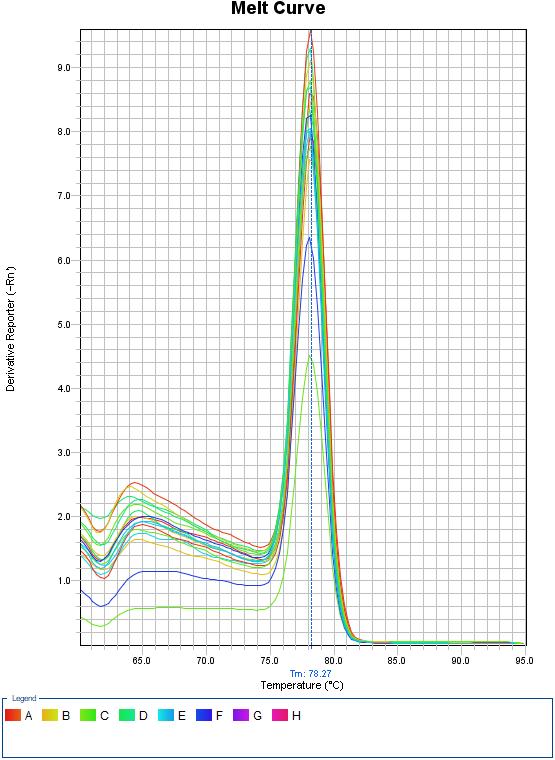


*Copia-17*


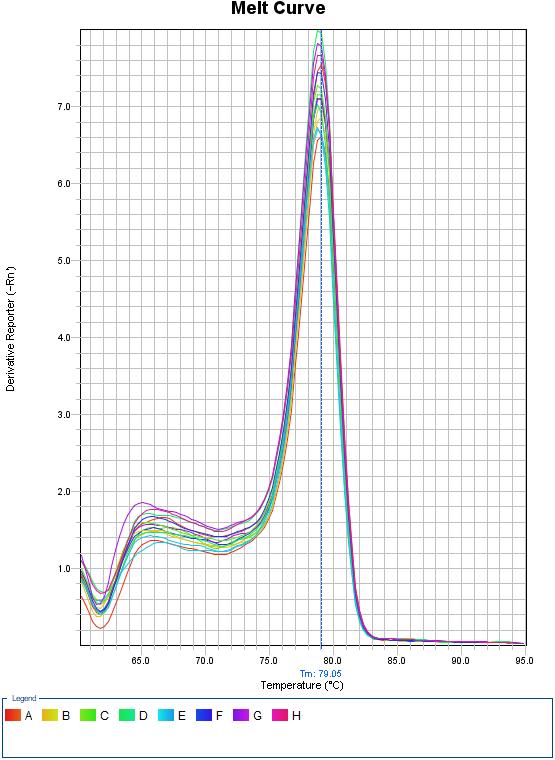


*Conagree*


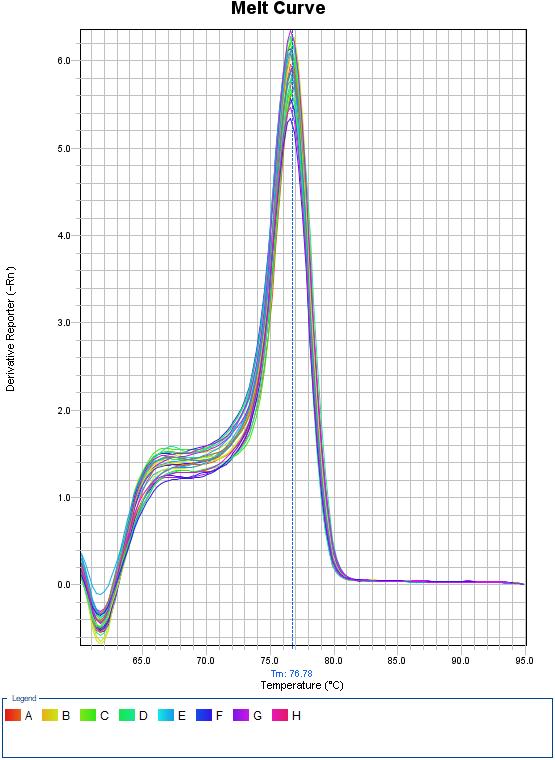


*Cumber-*

*land*


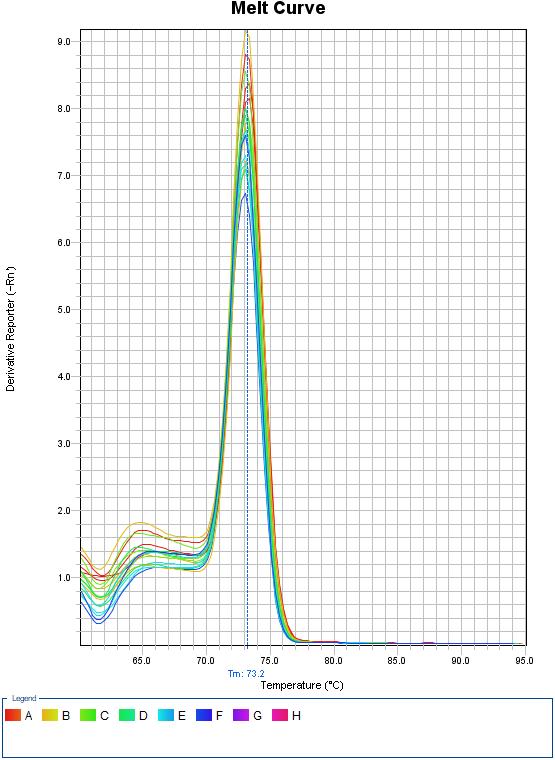


*IFG*


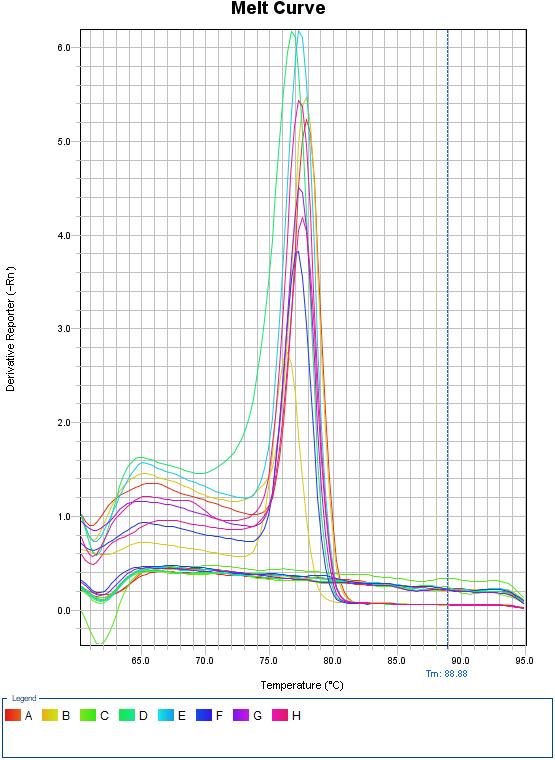


*Ozark**


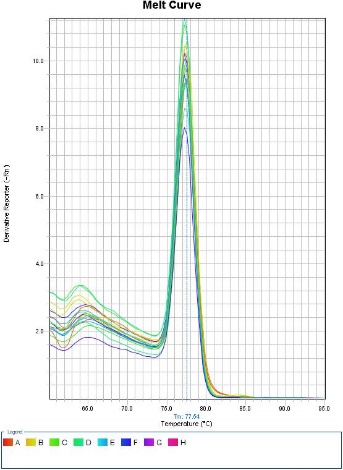


*Piney-*

*woods*


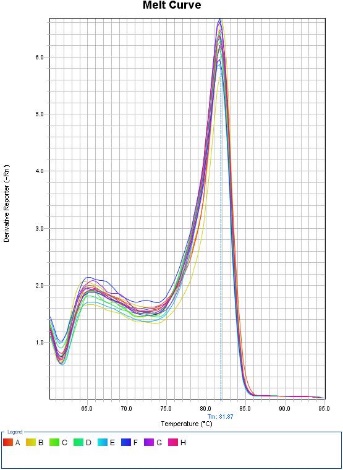


*Riga-4*


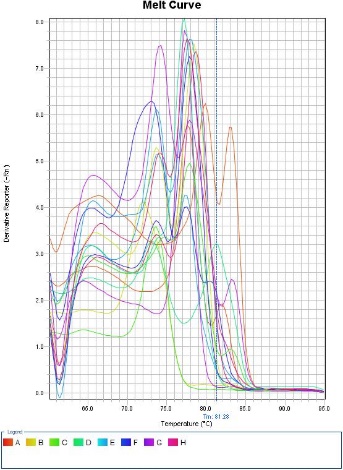


*Ouachita**


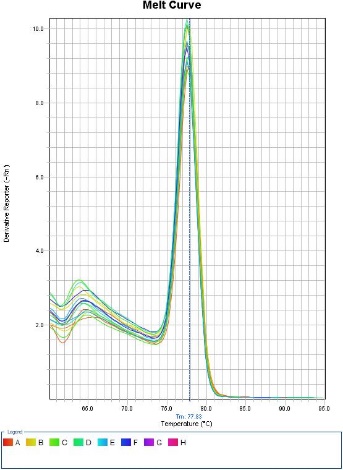


*Talladega*


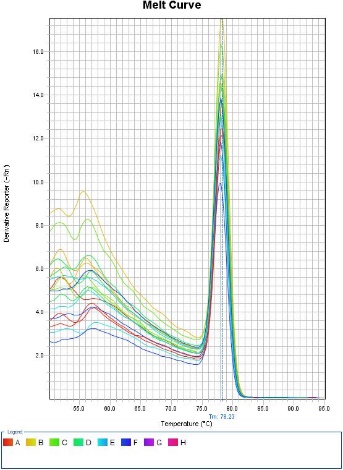


*Silava***

* The amplification product melting curves were non-specific, therefore these templates were not analysed further.

** Target not analysed further. The low copy number element *Silava* (4±0.47 copies in genome of *P.sylvestris* [20]) was present only in treated samples, the amplification curve displayed high CT levels, indicating low transcript abundance.
