# Supplementary material for: Retrotransposon expression in response to in vitro inoculation with two fungal pathogens of Scots pine (Pinus sylvestris L.)
Source: BMC Res Notes. 2019 Apr 29;12:243. doi: 10.1186/s13104-019-4275-3 (PMC6489336; doi:10.1186/s13104-019-4275-3)
Supplement: Supplementary file 6 — Additional file 6. a. Scatterplot matrix for pooled RE expression data correlation in needles after inoculation with LS. b. Scatterplot matrix for pooled RE expression data correlation in needles and roots after inoculation with HA. [file 13104_2019_4275_MOESM6_ESM.docx]

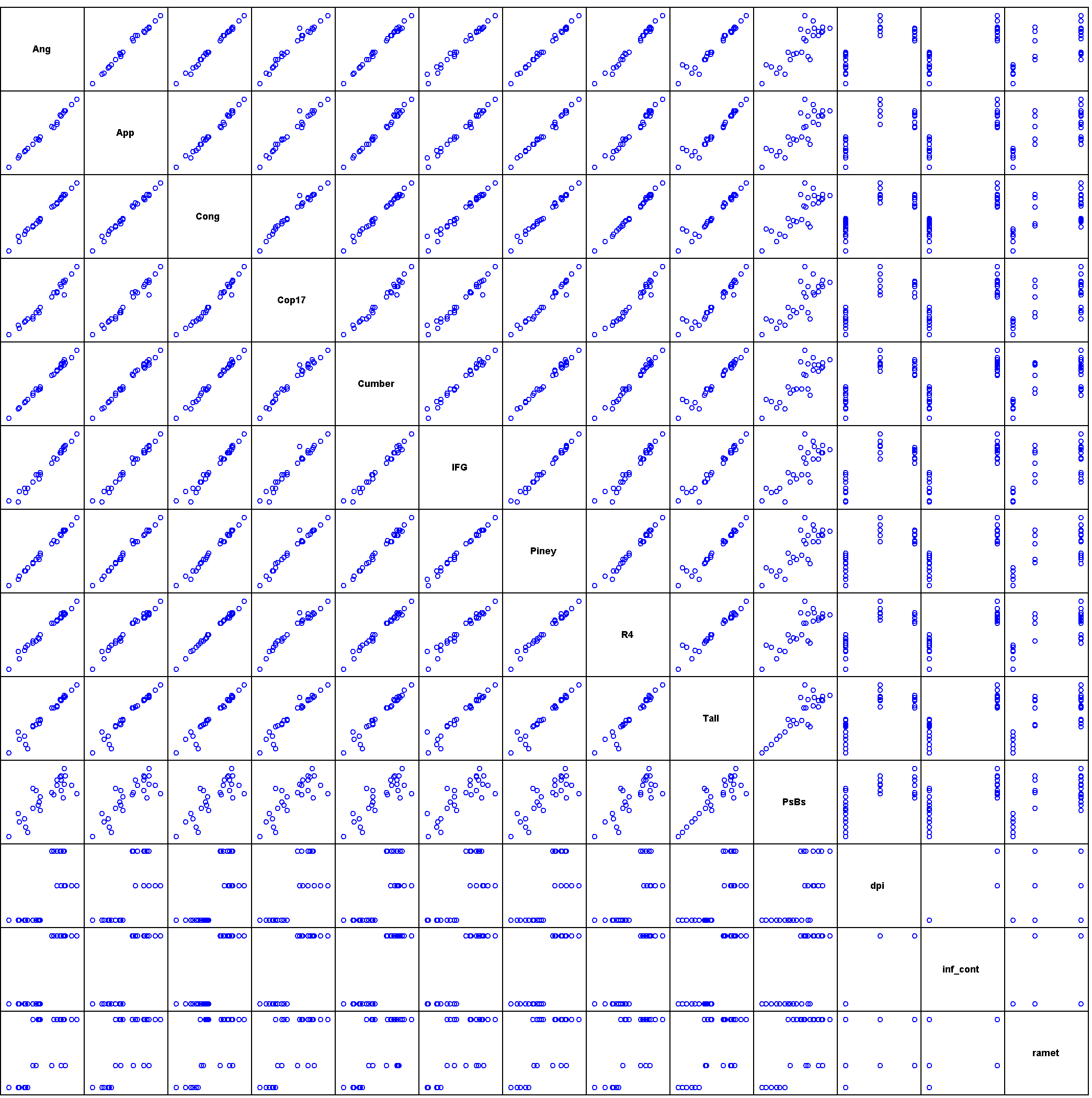


**Additional file 6a.** Scatterplot matrix for pooled RE expression data correlation in needles after inoculation with *L.seditiosum*. RE families (Cong (*Conagree*), R4 (*Riga-4*), Ang (*Angelina*), Cumb (*Cumberland*), App (*Appalachian*), Cop17 (*Copia-17-PTa*); IFG (*IFG-7a*); Piney (*Pineywoods*); Tall (*Talladega*), PsBs (*Pinosylvine synthetase* gene)). Categories used for grouping: dpi-days post inoculation; inf_cont- infected or control; ramet- pine ramet.


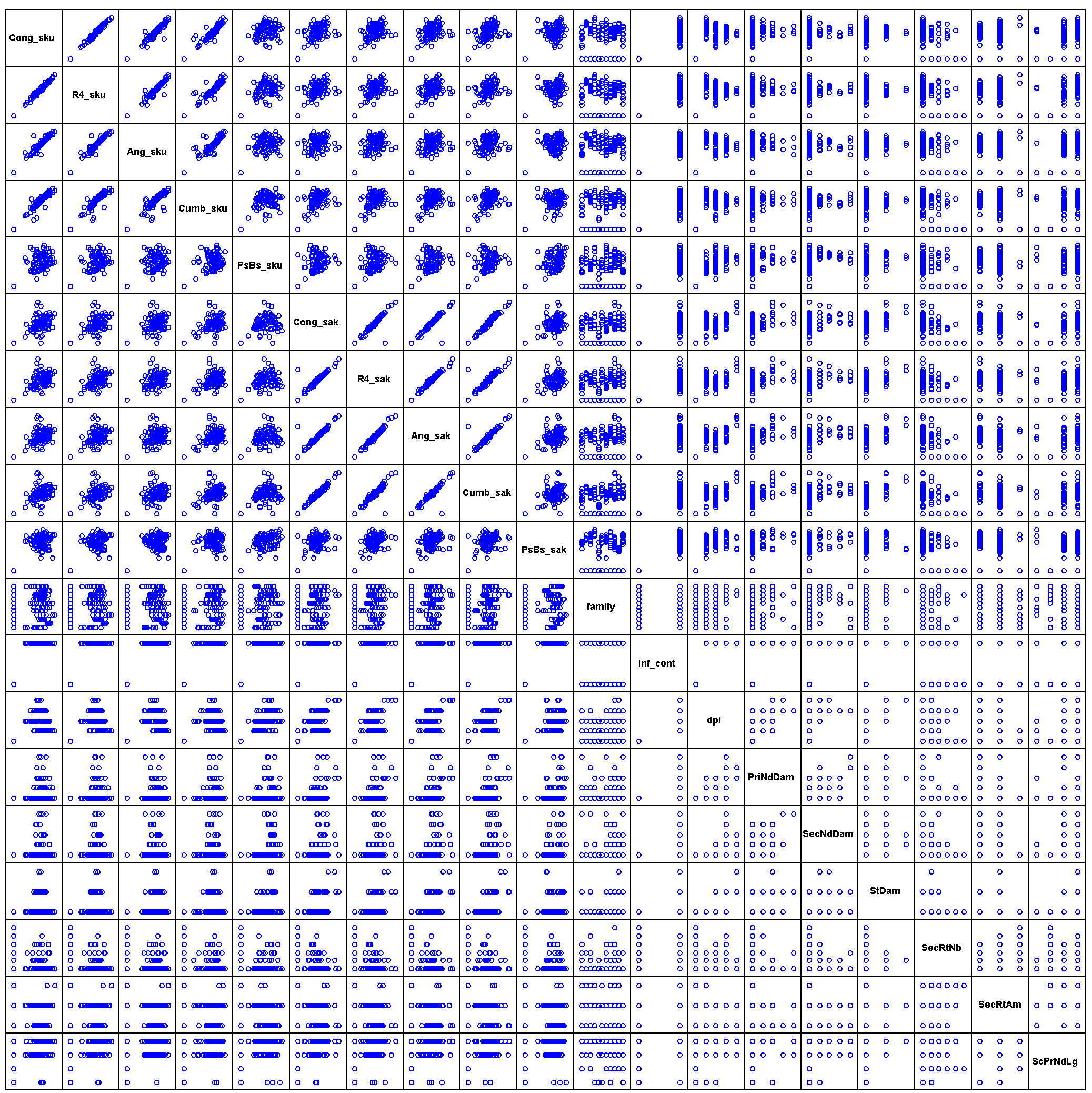


**Additional file 6b.** Scatterplot matrix for pooled RE expression data correlation in needles after inoculation with *H. annosum*. RE families (Cong (*Conagree*), R4 (*Riga-4*), Ang (*Angelina*), Cumb (*Cumberland*), PsBs (*Pinosylvine synthetase* gene)). SKU-Needles; SAK-Roots. Categories used for grouping: family- seedling family; inf_cont – infected or control; dpi- days post inoculation; PriNdDam- damage of primary needles; SecNdDam- damage of primary needles; StDam- damage of stem; SecRtNb- number of secondary rootlets; SecRtAm- amount of secondary rootlets; ScPrNdLg- secondary to primary needles length ratio.
