# Supplementary material for: Retrotransposon expression in response to in vitro inoculation with two fungal pathogens of Scots pine (Pinus sylvestris L.)
Source: BMC Res Notes. 2019 Apr 29;12:243. doi: 10.1186/s13104-019-4275-3 (PMC6489336; doi:10.1186/s13104-019-4275-3)
Supplement: Supplementary file 8 — Additional file 8. Summary of experimental group MRPP statistics. [file 13104_2019_4275_MOESM8_ESM.docx]

**Additional file 8.** Summary of experimental group MRPP statistics*. T: test statistic; A: chance-corrected within-group agreement; p: probability of a smaller or equal delta.

| **Group** | **Tissue** | **δ under null hypothesis** | | | | **T** | **p** | **A** |
| --- | --- | --- | --- | --- | --- | --- | --- | --- |
|  |  | **Observed δ** | **Expected δ** | **Variance** | **Skewness** |  |  |  |
| **Infection** | root | 0.2325 | 0.4599 | 0.68810841E-05 | -2.42 | -86.67 | 0.00000000 | 0.4943 |
|  | needle | 0.2703 | 0.4599 | 0.70307401E-05 | -2.24 | -71.48 | 0.00000000 | 0.4121 |
| **Treatment** | root | 0.2073 | 0.4599 | 0.29774968E-04 | -1.09 | -46.29 | 0.00000000 | 0.5492 |
|  | needle | 0.2303 | 0.4599 | 0.30422541E-04 | -1.00 | -41.62 | 0.00000000 | 0.4991 |
| **Seedling family** | root | 0.4418 | 0.4599 | 0.74323444E-04 | -0.69 | -2.09 | 0.03293225 | 0.0392 |
|  | needle | 0.4317 | 0.4599 | 0.75939897E-04 | -0.64 | -3.22 | 0.00450801 | 0.0611 |
| **Damage** | root | 0.4143 | 0.4599 | 0.69030618E-05 | -2.4 | -17.36 | 0.00000009 | 0.0991 |
|  | needle | 0.4271 | 0.4599 | 0.70531958E-05 | -2.24 | -12.33 | 0.00000380 | 0.0712 |
| **Damage**  **& infection** | root | 0.2236 | 0.4599 | 0.13804904E-04 | -1.70 | -63.60 | 0.00000000 | 0.5138 |
|  | needle | 0.2523 | 0.4599 | 0.14105146E-04 | -1.58 | -55.25 | 0.00000000 | 0.4512 |
| **Seedling family**  **& infection** | root | 0.3476 | 0.4599 | 0.17884583E-03 | -0.39 | -8.39 | 0.00000000 | 0.2441 |
|  | needle | 0.3678 | 0.4599 | 0.18273552E-03 | -0.35 | -6.80 | 0.00000010 | 0.2001 |

* Heterogeneity within the following groups was tested: infected vs. controls; treatment points (control, 7 dpi; 14 dpi; 21 dpi), family of seedlings (11), damage to primary needles, damage to secondary needles, damage to the stem, length of the secondary needles, number of lateral rootlets. Expression data were relativized before analyses. Distance matrix was rank-transformed. Sorensen (Bray-Curtis) distance measure was applied as it retains sensitivity in more heterogeneous data sets compared to Euclidean distance [31]. Significant differences were observed only between control and infected sample groups. Multiple pairwise comparisons of groups by treatment time also indicated significant difference between the control group and each sampling point after infection, while heterogeneity between groups taken after 7 dpi, 14 dpi, 21 dpi were similar to that expected by chance. Pooled group of plants with visually observed damage did not differ significantly by RE expression from group of infected plants without noticeable damage. However, separation of samples by seedlings family and infection revealed differences in RE response. Six pine families showed significant expression changes in the roots after inoculation (A=0.20-0.265), while two seedling families (Sm4, M236) displayed no significant changes (A<0.1) and M347, M259 and M248 showed a small change (A=0.1-0.16).
